# Long-term complete response of advanced hepatocellular carcinoma treated with multidisciplinary therapy including reduced dose of sorafenib: case report and review of the literature

**DOI:** 10.1186/s12957-015-0559-9

**Published:** 2015-04-09

**Authors:** Masahiro Shinoda, Norihiro Kishida, Osamu Itano, Shigenori Ei, Akihisa Ueno, Minoru Kitago, Yuta Abe, Taizo Hibi, Hiroshi Yagi, Yohei Masugi, Minoru Tanabe, Koichi Aiura, Michiie Sakamaoto, Akihiro Tanimoto, Yuko Kitagawa

**Affiliations:** Department of Surgery, School of Medicine, Keio University, 35 Shinanomachi, Shinjuku-ku, Tokyo 160-8582 Japan; Department of Pathology, School of Medicine, Keio University, 35 Shinanomachi, Shinjuku-ku, Tokyo 160-8582 Japan; Department of Diagnostic Radiology, School of Medicine, Keio University, 35 Shinanomachi, Shinjuku-ku, Tokyo 160-8582 Japan; Department of Surgery, Tokyo Medical and Dental University, 1-5-45 Yushima, Bunkyo-ku, Tokyo 113-8519 Japan; Department of Surgery, Kawasaki Municipal Hospital, 12-1 Shinkawadori, Kawasaki-shi, Kawasaki-ku 210-0013 Japan

**Keywords:** Hepatocellular carcinoma, Tumor thrombi, Sorafenib, Reduced dose, Long-term complete response

## Abstract

An 83-year-old man underwent computed tomography during a routine check-up due to a history of surgical treatment for pancreatic cancer. Two tumors were detected in the anterior segment of the liver. A needle biopsy of the larger tumor was performed, and pathological examination showed that the tumor was a poorly differentiated hepatocellular carcinoma. Resection was not performed considering the patient’s poor physical condition. Thus, transcatheter arterial chemoembolization and radiofrequency ablation of the tumors were performed. Three months later, residual tumor of the larger lesion and multiple pulmonary metastases were detected. This time, continuous hepatic arterial infusion chemotherapy was performed. Although the pulmonary metastases markedly reduced, tumor thrombi appeared in the right portal vein on computed tomography. Finally, sorafenib was administered, which led to disappearance of the tumor thrombi and no other signs of recurrence 8 months after initiation of sorafenib on computed tomography. Although sorafenib administration has continued at reduced doses of 200 mg per day or less due to hypertension, complete response has persisted for the past 34 months. It is noteworthy that sorafenib has been given at reduced doses, but a long-term complete response is maintained in a patient who had portal tumor thrombi and distant metastasis. Herein, we present this rare case of advanced hepatocellular carcinoma controlled with reduced doses of sorafenib following multidisciplinary therapy, describe our single center experience with sorafenib use in patients with hepatocellular carcinoma, and review previous reports that focused on dose reduction of sorafenib.

## Background

Although potentially curative therapies including liver transplantation, resection, and ablations have been developed for hepatocellular carcinoma (HCC), patients with advanced stage of HCC still have poor prognosis [1-3]. The recent development of molecular targeted therapies has begun to change strategies for treating advanced HCC. The efficacy and safety of sorafenib for advanced HCC were assessed in the SHARP study [4] and also in the Asia-Pacific study [5]. To date, sorafenib has been applied to a large number of patients worldwide and becomes an important therapeutic option for HCC. A growing number of complete response (CR) patients have been reported since 2008 [6]. Recently, a Japanese multi-center study has reported 18 patients with HCC who achieved CR after sorafenib treatment and precisely described their characteristics [7]. However, it is still very difficult and extremely rare to maintain CR for long term in patients with advanced HCC. We herein report a case of advanced HCC that was initially treated with multidisciplinary therapy and has maintained a CR status for 34 months using reduced doses of sorafenib. We also describe our single-center experience for sorafenib use in patients with HCC and review previous reports that focused on HCC patients with long-term CR using reduced doses of sorafenib.

## Case presentation

**Figure 1:**
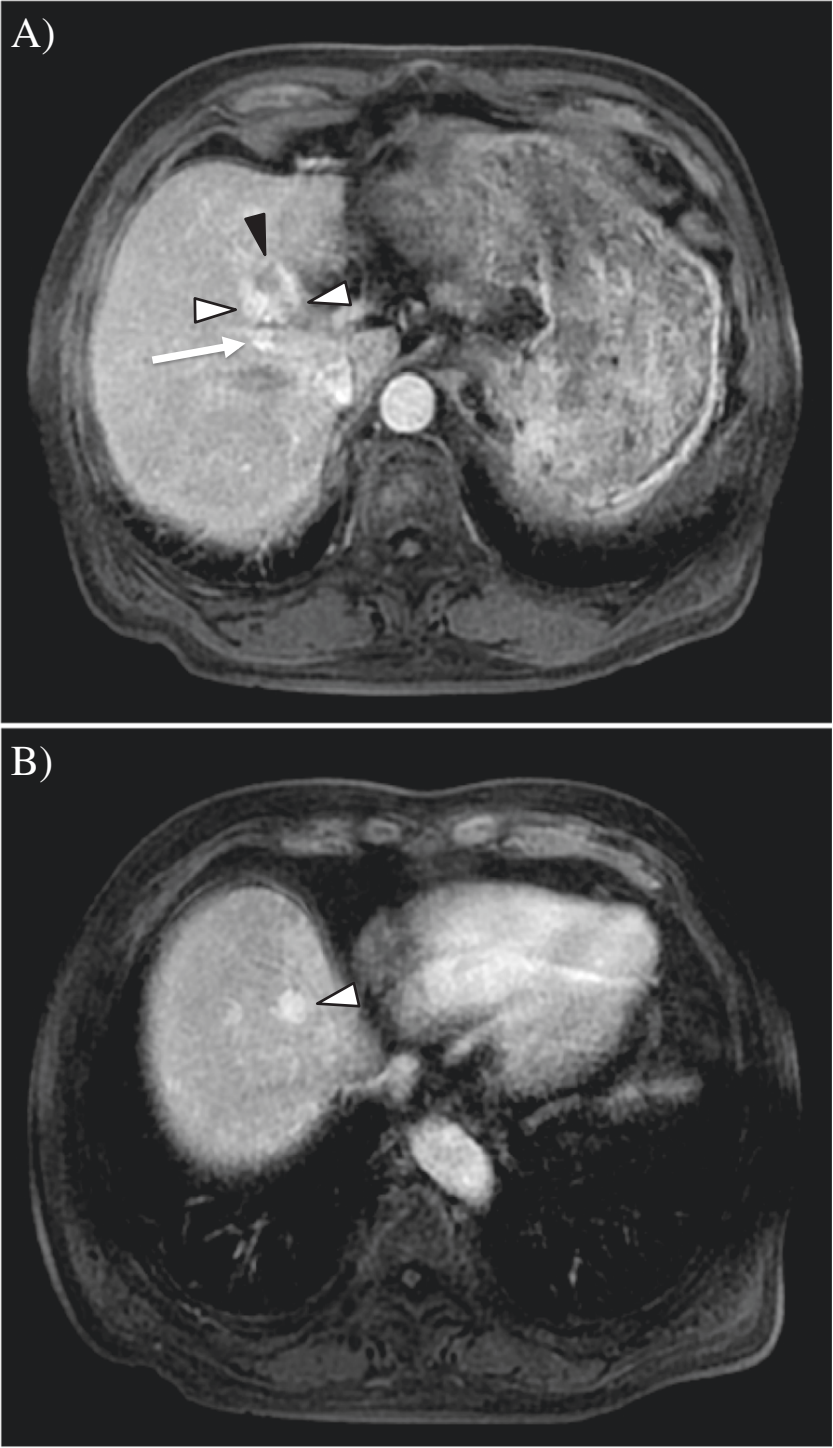
**Gadoxetic acid-enhanced magnetic resonance imaging at tumor discovery.** Images in the arterial-phase are shown. **(A)** A large tumor, 23 mm in diameter, is adjacent to the right Glisson branch (white arrow). The dorsal side of the tumor is hypervascular (early enhancement is indicated by white triangles), and the ventral side is hypovascular (indicated by a black triangle). **(B)** A smaller tumor, 13 mm in diameter, is below the right diaphragm. The tumor also shows early enhancement (indicated by a white triangle).

**Figure 2:**
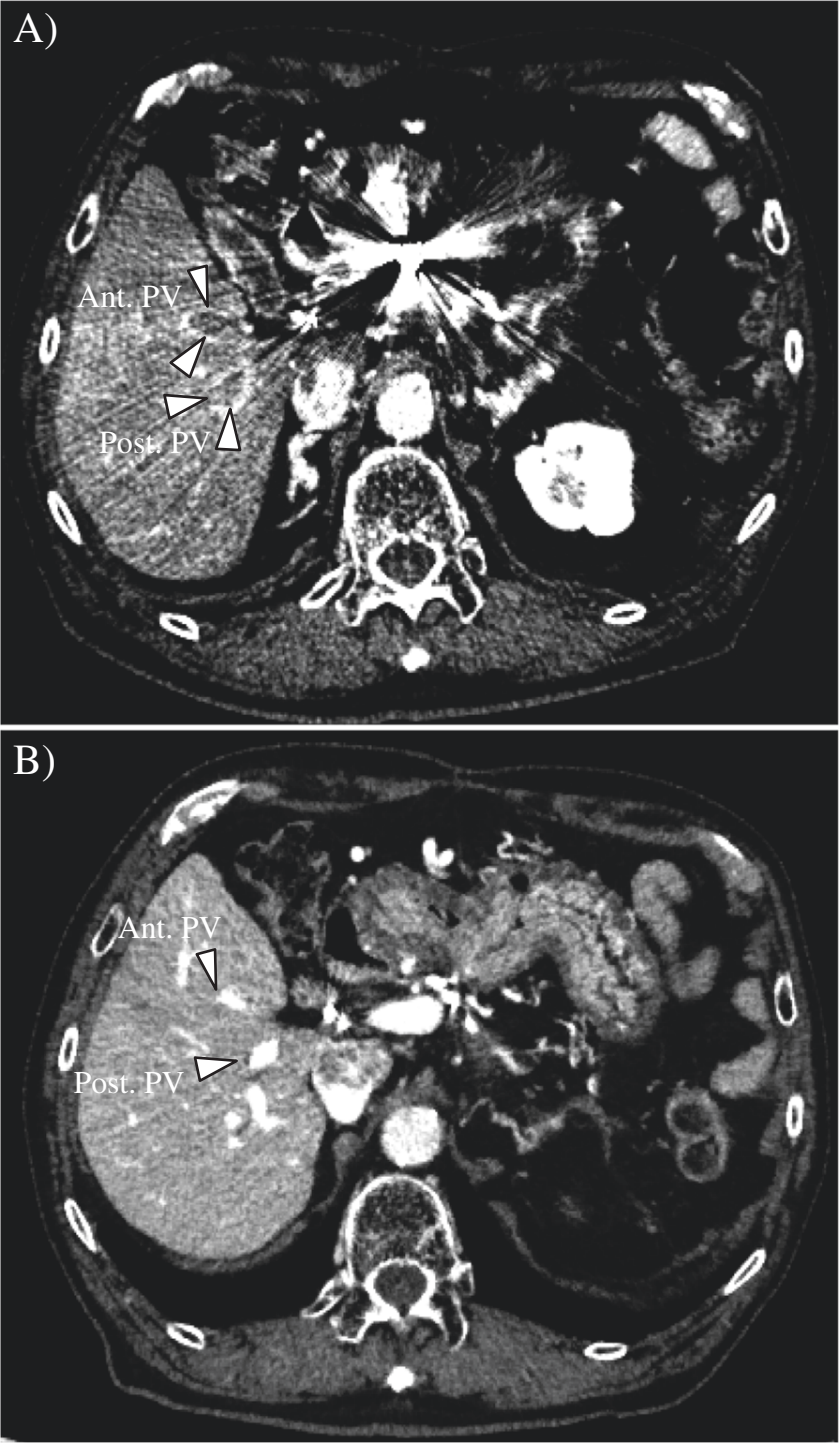
**Abdominal contrast-enhanced computed tomography before and after sorafenib introduction. (A)** *Before sorafenib introduction*. Massive filling defects with enhancement are seen in the anterior and posterior branches of the portal vein (indicated by white triangles). An artifact by the catheter and coil placed for hepatic arterial infusion chemotherapy is seen. **(B)** *After sorafenib introduction*. The tumor thrombi disappeared in the portal vein (indicated by white triangles). Ant. PV, anterior branch of the portal vein; Post. PV, posterior branch of the portal vein.

**Figure 3:**
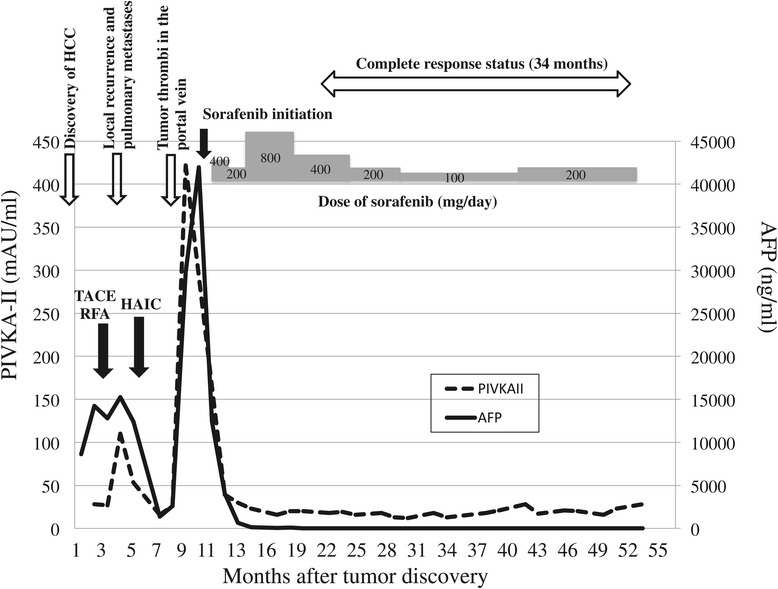
**Clinical course as assessed by tumor markers and therapeutic events from discovery of hepatocellular carcinoma to present.** HCC, hepatocellular carcinoma; TACE, transcatheter arterial chemoembolization; RFA, radiofrequency ablation; HAIC, hepatic arterial infusion chemotherapy; AFP, alpha-fetoprotein.

An 83-year-old Japanese man underwent distal pancreatectomy for pancreatic body cancer 3 years ago. He had a medical history of hypertension but not of hepatitis virus infection and alcohol abuse. During a postoperative surveillance examination, contrast-enhanced computed tomography (CT) detected two tumors, 23 and 13 mm in diameter, in the anterior segment of the liver. The larger tumor was adjacent to the right main Glisson branch, and the other lesion was below the right diaphragm. Both of them showed early enhancement in the arterial phase (Figure 1A,B) and hypointensity in the hepatobiliary phase in the gadoxetic acid-enhanced magnetic resonance imaging. The patient’s serum alpha-fetoprotein (AFP) level was elevated to 8,650 ng/ml (normal level is less than 20 ng/ml). A needle biopsy of the larger tumor was performed, and pathological examination showed that the tumor was a poorly differentiated hepatocellular carcinoma. A specimen from the previously diagnosed pancreatic cancer was re-examined, but the finding of ductal adenocarcinoma was identical to the one diagnosed at the time of surgery; and components of hepatoid carcinoma were not found in this specimen. Resection was not performed considering the tumor location (right lobectomy was necessary) and the patient’s hepatic functional reserve (Child-Pugh classification was grade A, but indocyanine green retention rate at 15 min was 21%) and advanced age. Since the larger tumor was adjacent to the right main Glisson branch and only the bordering side appeared hypervascular, we first performed transcatheter arterial chemoembolization to the hypervascular part of the larger tumor and to the other tumor and then additionally performed radiofrequency ablation of the hypovascular portion of the larger tumor. Three months after the treatment, CT imaging revealed a residual tumor of the larger lesion at the ablation site and multiple lung metastases. Thus, continuous hepatic arterial infusion chemotherapy using 5-fluorouracil and cisplatin was performed for 2 months. This was effective in reducing the residual tumor and lung metastases, but tumor thrombi appeared in the right portal vein (Figure 2A), and his AFP and PIVKA-II levels were elevated to 41,948 ng/ml and 422 mAU/mL, respectively (Figure 3). Subsequently, sorafenib was administered at a reduced dose of 400 mg per day considering his advanced age (the manufacturer’s recommended dose is 800 mg per day). The dose of sorafenib was temporarily increased to 800 mg per day but was reduced to 400 mg per day again due to an adverse event of hypertension (grade 3 in Common Terminology Criteria for Adverse Effects version 4.0) (doses are described in Figure 3). Eight months after initiation of sorafenib, his AFP level was decreased to normal level, and both the tumor thrombi in the right portal vein and multiple lung metastases disappeared on CT imaging (Figure 2B). The dose of sorafenib was further reduced to 200 mg per day or 200 mg per 2 days due to hypertension, but there have been no signs of recurrence on CT imaging; and his AFP and PIVKA-II levels have also been within the normal limits for the past 34 months.

## Discussion

This patient underwent multidisciplinary therapy including transcatheter arterial chemoembolization, radiofrequency ablation, and continuous hepatic arterial infusion chemotherapy until tumor thrombi were found in the portal vein. We finally initiated sorafenib treatment, and the patient achieved CR soon afterwards and has maintained the CR status for the past 34 months. Based on the clinical course of this patient, the achievement of CR was predominantly attributed to sorafenib therapy. It is extremely rare that a patient with advanced HCC and portal tumor thrombi can achieve long-term CR without resection.

According to the published literature, there were only four cases of HCC who achieved CR using mainly sorafenib and maintained the status for more than 2 years [8-10]. Table 1 summarizes the patient characteristics of the four reported cases and the present case. All patients were male, and the mean age was 73.4 years old. The background liver disease was hepatitis C in three cases, hepatitis B in one case, and nothing in one case (present case). The extent of HCC was major vascular invasion in three cases, distant metastasis in one case, and both major vascular invasion and distant metastasis in one case (present case). It is noteworthy that sorafenib was initiated at the recommended dose (800 mg per day) in three cases, but the dose was soon reduced to 400 mg per day or less in all of them. The final maintenance dose of sorafenib was 400 mg per day in the four reported cases and 200 mg per day in the present case, although the SHARP [4] and Asia-Pacific [5] studies reported that efficacy of sorafenib for advanced HCC was maximally observed at the dose of 800 mg per day.

**Table 1:** **Cases who achieved complete response for more than 2 years in the literature**

	**Case 1**	**Case 2**	**Case 3**	**Case 4**	**Present case**
Reported year [reference]	2011 [8]	2011 [8]	2013 [9]	2013 [10]	2014
Age/sex	70/male	69/male	71/male	74/male	83/male
Background liver disease	Hepatitis C	Hepatitis C	Hepatitis C	Hepatitis B	Non-viral, non-alcoholic
Tumor status at sorafenib initiation					
Number	Single	Not described	Multiple	Single	-
Location	Not described	Three hepatic lesions	Segment IV and VII	Posterior segment	Right lobe
Maximum size	6 × 5 cm	Not described	9 cm	8.6 × 5.7 cm	-
Extension	Vascular invasion	Portal vein thrombosis	Portal vein thrombosis	Vertebral metastasis	Lung metastasis
Pathological diagnosis	None (based on the computed tomography image)	None (based on the computed tomography image)	Poorly/moderately differentiated hepatocellular carcinoma	Moderately differentiated hepatocellular carcinoma	Poorly differentiated hepatocellular carcinoma
Pretreatment before sorafenib	None	Not described	Synchro-level, Vitamin E	TACE → PEIT, RFA	TACE + RFA → HAIC (5-FU + CDDP)
Doses of sorafenib (period)					
Initial dose	Not described	800 mg/day (10 days)	800 mg/day (2 months)	800 mg/day (2 weeks)	400 mg/day (1 month)
Second dose	→ 400 mg/day (55 months)	→ 400 mg/day (3 months)	→ 400 mg/day (18 months)	→ 400 mg/day (60 months)	→ 200 mg/day (1 months)
Third dose		→ 400 mg/2 days (57 months)			→ 800 mg/day (4 months)
Fourth dose					→ 400 mg/day (2 months)
Last doses					→ 200 → 100 → 200 mg/day (34 months)
Total period of sorafenib	60 months	62 months	20 months	60 months	42 months
Period of complete response	<27 months	38 months	28 months	54 months	34 months
Reported status	Alive	Alive	Alive	Alive	Alive

TACE, transcatheter arterial chemoembolization; RFA, radiofrequency ablation; PEIT, percutaneous ethanol injection therapy; HAIC, hepatic arterial infusion chemotherapy; 5-FU, 5-fluorouracil; CDDP, cisplatin.

**Figure 4:**
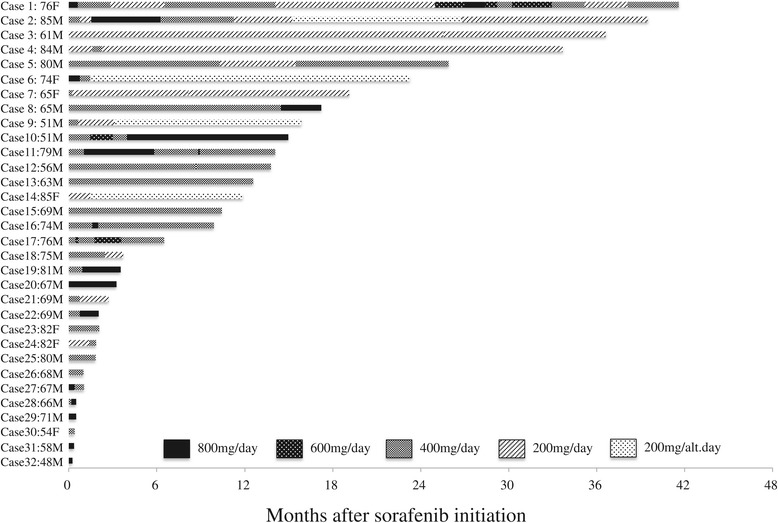
**List of cases treated with sorafenib for hepatocellular carcinoma in our department and the doses of sorafenib.** Cases are shown in descending order of length of sorafenib treatment. Case 2 represents the present case. Ages shown are at the time of sorafenib initiation.

We also surveyed our single department with regard to sorafenib use for advanced HCC. In 2010, sorafenib became commercially available for advanced HCC in Japan and has been administered to 32 patients with advanced HCC so far in our department. We applied sorafenib at the recommended dose of 800 mg per day in seven patients (cases 1, 6, 20, 27, 29, 31, and 32 in Figure 4), but all of the patients did not tolerate this recommended dose due to side effects or disease progression. Thus, the dose had to be reduced in two cases (cases 1 and 6 in Figure 4), and the treatment itself was discontinued in five cases (cases 20, 27, 29, 31, and 32 in Figure 4) within 3 months after initiation of sorafenib. We also found that 14 cases (cases 1 to 14 in Figure 4) took sorafenib for more than 12 months and 7 cases (cases 1 to 7 in Figure 4) took sorafenib for more than 18 months. Most of the long-term recipients of sorafenib were not given the recommended dose of 800 mg per day and had instead reduced doses for most of the treatment period.

## Conclusion

We present here a rare case of advanced HCC, who was initially treated with multidisciplinary therapy including transcatheter arterial chemoembolization, radiofrequency ablation, and continuous hepatic arterial infusion chemotherapy, and achieved long-term CR using reduced doses of sorafenib. Based on our single-center experience of sorafenib use in patients with HCC and the previously reported cases of long-term CR using sorafenib, it appears that it is important to continue sorafenib for a long time even if the dose is reduced. We hope that this case presentation and literature review serves as a stimulus for further investigation to clarify if using reduced doses of sorafenib for maintenance increase the incidence of CR.

## Consent

Written informed consent was obtained from the patient for the publication of the case report and accompanying images. This case report was written in accordance with the Declaration of Helsinki following approval from our Institutional Review Board (authorization number, 20120443).

## Abbreviations

HCC: hepatocellular carcinoma
CR: complete response
CT: computed tomography
AFP: alpha-fetoprotein
